# Initiation of Pancreatic Cancer: The Interplay of Hyperglycemia and Macrophages Promotes the Acquisition of Malignancy-Associated Properties in Pancreatic Ductal Epithelial Cells

**DOI:** 10.3390/ijms22105086

**Published:** 2021-05-11

**Authors:** Lilli Otto, Sascha Rahn, Tina Daunke, Frederik Walter, Elsa Winter, Julia Luisa Möller, Stefan Rose-John, Daniela Wesch, Heiner Schäfer, Susanne Sebens

**Affiliations:** 1 Institute for Experimental Cancer Research, Kiel University (CAU) and University Medical Center Schleswig-Holstein (UKSH), Campus Kiel, 24105 Kiel, Germany; lilli.otto@web.de (L.O.); tina.daunke@email.uni-kiel.de (T.D.); frederikwalter@yahoo.com (F.W.); elsa.winter@mail.de (E.W.); hschaef@1med.uni-kiel.de (H.S.); 2 Institute of Biochemistry, Kiel University, 24118 Kiel, Germany; srahn@biochem.uni-kiel.de (S.R.); rosejohn@biochem.uni-kiel.de (S.R.-J.); 3 Department of Hematology and Oncology, University Medical Center Schleswig-Holstein (UKSH) Campus Kiel, 24105 Kiel, Germany; luisafaltinek@gmail.com; 4 Institute of Immunology, Kiel University and University Medical Center Schleswig-Holstein (UKSH) Campus Kiel, 24105 Kiel, Germany; Daniela.Wesch@uksh.de

**Keywords:** pancreatic cancer, tumor microenvironment, hyperglycemia, macrophages, EMT, type 2 diabetes, cell migration

## Abstract

Pancreatic ductal adenocarcinoma (PDAC) is still one of the most aggressive solid malignancies with a poor prognosis. Obesity and type 2 diabetes mellitus (T2DM) are two major risk factors linked to the development and progression of PDAC, both often characterized by high blood glucose levels. Macrophages represent the main immune cell population in PDAC contributing to PDAC development. It has already been shown that pancreatic ductal epithelial cells (PDEC) undergo epithelial–mesenchymal transition (EMT) when exposed to hyperglycemia or macrophages. Thus, this study aimed to investigate whether concomitant exposure to hyperglycemia and macrophages aggravates EMT-associated alterations in PDEC. Exposure to macrophages and elevated glucose levels (25 mM glucose) impacted gene expression of EMT inducers such as IL-6 and TNF-α as well as EMT transcription factors in benign (H6c7-*pBp*) and premalignant (H6c7-*kras*) PDEC. Most strikingly, exposure to hyperglycemic coculture with macrophages promoted downregulation of the epithelial marker E-cadherin, which was associated with an elevated migratory potential of PDEC. While blocking IL-6 activity by tocilizumab only partially reverted the EMT phenotype in H6c7-*kras* cells, neutralization of TNF-α by etanercept was able to clearly impair EMT-associated properties in premalignant PDEC. Altogether, the current study attributes a role to a T2DM-related hyperglycemic, inflammatory micromilieu in the acquisition of malignancy-associated alterations in premalignant PDEC, thus providing new insights on how metabolic diseases might promote PDAC initiation.

## 1. Introduction

Pancreatic ductal adenocarcinoma (PDAC) is the seventh most frequent cause of cancer related deaths worldwide with the highest rates in Western countries [1]. Eighty percent of all PDAC patients are first diagnosed in late stages of tumorigenesis, implying a low overall 5-year survival rate of less than ten percent [2,3,4]. In addition to higher age and lifestyle factors such as smoking or alcohol abuse, a long-standing chronic pancreatitis (CP) is considered to play a major role in PDAC development [5,6]. Further risk factors associated with the development and progression of PDAC are obesity and type 2 diabetes mellitus (T2DM), which are both often characterized by elevated blood glucose levels [5,7,8]. In this context, recent studies and meta-analyses showed that individuals with long-term T2DM are exposed to a 2-fold increased risk of PDAC onset compared with non-diabetic individuals [7] and that with every 0.56 mmol/l increase in fasting blood glucose levels, PDAC risk is elevated by 14% [9]. However, it is still poorly understood how hyperglycemia promotes PDAC initiation. 

A recent study showed that a high fat diet in association with oncogenic *Kras* activation, which is the earliest genetic alteration in PDAC development and is already found in low-grade pancreatic intraepithelial neoplasia (PanIN), significantly increases the formation of pancreatic tumors [10].

Epithelial–mesenchymal-transition (EMT) is a process enabling carcinoma cells to acquire a mesenchymal phenotype and thereby allowing them to leave the primary context, disseminate and seed at secondary sites [11]. Additionally, EMT has been closely linked to cancer stemness as it could be shown that transforming growth factor-beta1 (TGF-β1) concomitantly induces EMT and stemness-associated properties in breast cancer cells [12]. Both processes are regulated by common transcription factors such as Snail, Slug or Zeb1 [13].

Cancer stem cells (CSC) are not only essential for the initiation and progression of cancer but also for recurrences and therapy failure due to their unique self-renewal capacity, their ability to give rise to differentiated daughter cells and their profound survival strategies [12,14,15]. These and other studies also indicate that EMT and CSC properties can be gained and lost in a context-dependent manner [16,17].

In a genetically engineered mouse model, Rhim et al. demonstrated that pancreatic epithelial cells harboring oncogenic *Kras* mutation undergo EMT and intravasate from low-grade PanIN into the blood stream followed by dissemination to the liver already prior to primary tumor formation [13]. Moreover, inflammation of the pancreas aggravated EMT induction and dissemination of PanIN cells, underscoring the impact of inflammation on pancreatic tumorigenesis and in particular to EMT-associated processes [13].

Macrophages are key players in cancer-driving inflammation as they already accumulate in precancerous lesions and promote the acquisition of many cancer hallmarks, such as the ability of invasion and metastasis [18,19]. Our own data demonstrate that macrophages are the most abundant immune cell population in CP as well as in PDAC and that they essentially promote EMT and cell invasion of premalignant and malignant pancreatic ductal epithelial cells (PDEC) [20,21]. Moreover, we could recently show that PDEC acquire CSC properties along with a mesenchymal phenotype when exposed to a hyperglycemic concentration of 25 mM d-glucose [22]. Similar observations have been reported for other tumor entities [23,24].

It has also been shown that glucose plays a central role in the differentiation of macrophages into a pro-inflammatory M1 phenotype [25]. Hence, exposure of murine macrophage cell lines but also of human macrophages to high glucose concentrations promotes a pro-inflammatory M1 phenotype [26,27,28]. Metabolic diseases such as obesity and T2DM are associated with a chronic state of inflammation, which is predominantly driven by macrophages. Accordingly, it could be demonstrated that hyperglycemia, a key feature of these two metabolic disorders, promotes macrophage-driven inflammation in adipose tissues [29]. However, it is still poorly understood whether hyperglycemia as manifested in prediabetic/diabetic individuals further aggravates the dangerous interplay of macrophages and PDEC, thereby fostering pancreatic tumorigenesis. The present study aimed at gaining further insights into the mechanisms of how inflammatory conditions associated with metabolic disorders promote malignancy-associated alterations in PDEC.

## 2. Results

We could already show that macrophages, which are abundant in CP and PDAC tissues [21], promote EMT and enhance cell motility in benign and malignant PDEC [20]. Moreover, we provided evidence that hyperglycemia is also able to induce EMT, along with CSC properties in these cells [22]. Expanding these findings, the present study aimed at investigating whether concomitant exposure to macrophages and hyperglycemia further promotes EMT- and CSC-associated alterations in PDEC. To mimic short- as well as long-term effects, benign H6c7-*pBp* and premalignant H6c7-*kras* cells were cultured alone or in direct coculture with M1 macrophages, under normo- (5 mM d-glucose) or hyperglycemic (25 mM d-glucose) conditions for 2 or 5 days, respectively.

### 2.1. The Presence of Macrophages as well as Hyperglycemia Impacts mRNA Levels of EMT and CSC Inducers in PDEC

**Figure 1:**
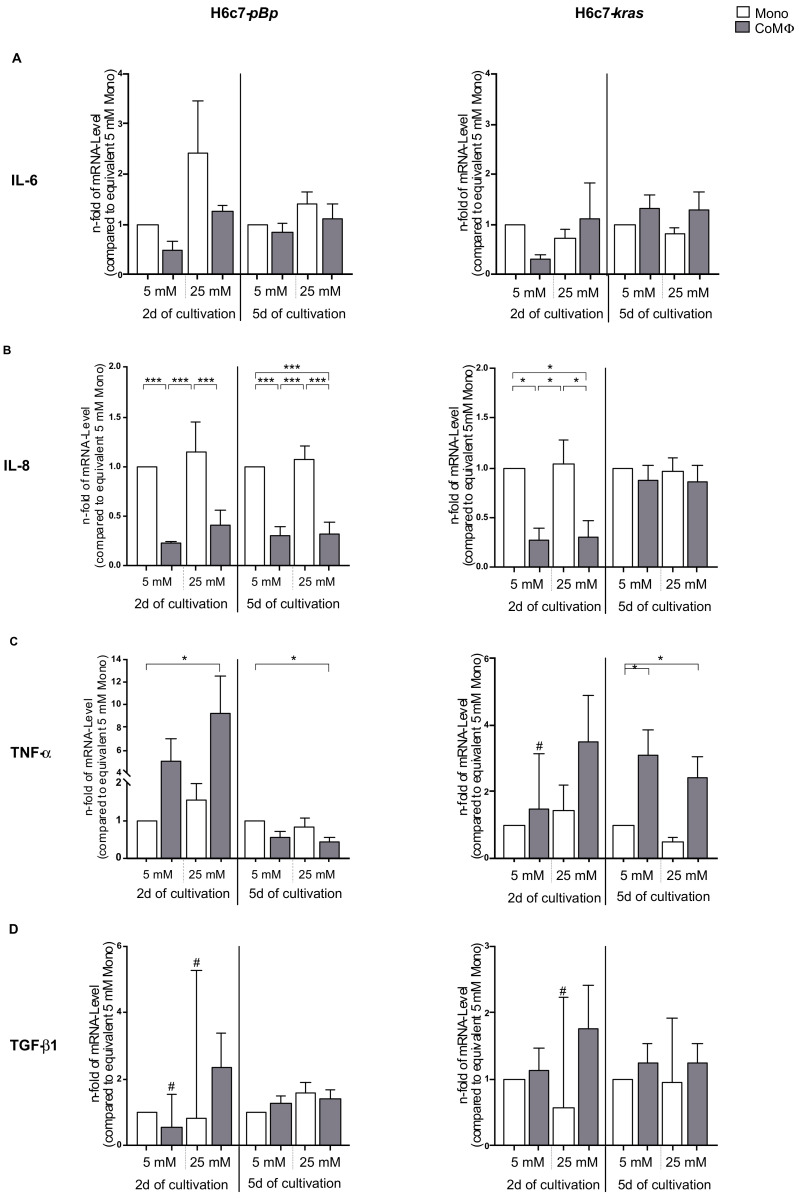
The presence of macrophages and hyperglycemia impacts mRNA levels of EMT and CSC inducers in PDEC. H6c7-*pBp* or H6c7-*kras* cells were cultivated in mono- or coculture with M1-polarized macrophages (MΦ) under normo- or hyperglycemic conditions (5 or 25 mM of d-glucose) for 2 or 5 days. The epithelial cells were separated from direct coculture with macrophages via CD11b-MACS depletion of M1-MΦ or harvested from monoculture and used for qRT-PCR analysis. The relative mRNA levels of IL-6 (**A**), IL-8 (**B**), TNF-α (**C**) and TGFβ-1 (**D**) for both cell lines are depicted. They are normalized to the housekeeping gene GAPDH and presented as *n*-fold expression compared with the monocultured 5 mM sample from the equivalent cultivation timespan. Normally distributed data are presented as mean and standard error of mean; not normally distributed data (indicated via #) are shown as median and interquartile range. * 0.05 > *p* > 0.0332; *** 0.002 > *p* > 0.0001; *n* = 4 for 2-day culture; *n* = 7 for 5-day culture.

First of all, we analyzed mRNA levels of EMT and CSC inducers—namely, the cytokines IL-6, IL-8, TNF-α and TGFβ-1—in PDEC exposed to M1-polarized macrophages and/or hyperglycemia. As seen in Figure 1, the presence of M1 macrophages as well as hyperglycemia altered the cytokine mRNA levels in PDEC, albeit cell-line specific differences could be determined. In this context it was also notable that the basal mRNA levels of all inflammatory mediators were higher in H6c7-*kras* cells than in H6c7-*pBp* cells after 2 days of normoglycemic monoculture (Figure S1). 

IL-6 mRNA levels were slightly increased in H6c7-*pBp* cells by hyperglycemia, whereas the presence of macrophages led to decreased mRNA levels under normoglycemic conditions after 2 days. In H6c7-*kras* cells, the presence of macrophages had a stronger effect on IL-6 expression than hyperglycemia. While short-term exposure to macrophages in normoglycemia led to a 0.3-fold reduction in premalignant PDEC, long-term exposure to macrophages resulted in increased IL-6 mRNA levels irrespective of the glucose concentration (Figure 1A). IL-8 mRNA levels were not affected by hyperglycemia in either cell line; however, a significant reduction could be observed in H6c7-*pBp* cells when cocultured with macrophages at both time points, ranging between 0.37- and 0.23-fold under both glucose concentrations. In H6c7-*kras* cells, on the other hand, a significant macrophage-associated reduction of IL-8 mRNA levels could only be observed after 2 days of coculture (Figure 1B).

Interestingly, TNF-α mRNA levels were increased in the benign PDEC by both coculture with macrophages and hyperglycemia after short-term culture (reaching a 9.24-fold increase in hyperglycemic coculture compared with normoglycemic monoculture), while this effect was not visible anymore after 5-day culture. Here, concomitant exposure to macrophages and hyperglycemia led to a 0.44-fold reduction compared with normoglycemic H6c7-*pBp* monoculture. In premalignant H6c7-*kras* cells, a clear coculture-dependent increase could be observed at both time points. This increase was most prominent after normoglycemic 5-day exposure to macrophages, leading to a significant 3.1-fold elevation compared with the equivalent normoglycemic cultured cells (Figure 1C). Finally, mRNA levels of TGFβ-1 were almost not affected in H6c7-*pBp* cells and slightly enhanced after coculture with macrophages predominantly in H6c7-*kras* cells after short-term culture, while hyperglycemia had not effect (Figure 1D).

Altogether, these data indicate that the interplay of PDEC with macrophages and hyperglycemia impacts mRNA levels of inflammatory EMT and CSC inducers in both PDEC populations; however, superior effects of concomitant exposure to both factors could only be observed for TNF-α.

### 2.2. Combined Impact of the Presence of M1 Macrophages and Hyperglycemia on mRNA Levels of EMT and CSC Transcription Factors in PDEC

**Figure 2:**
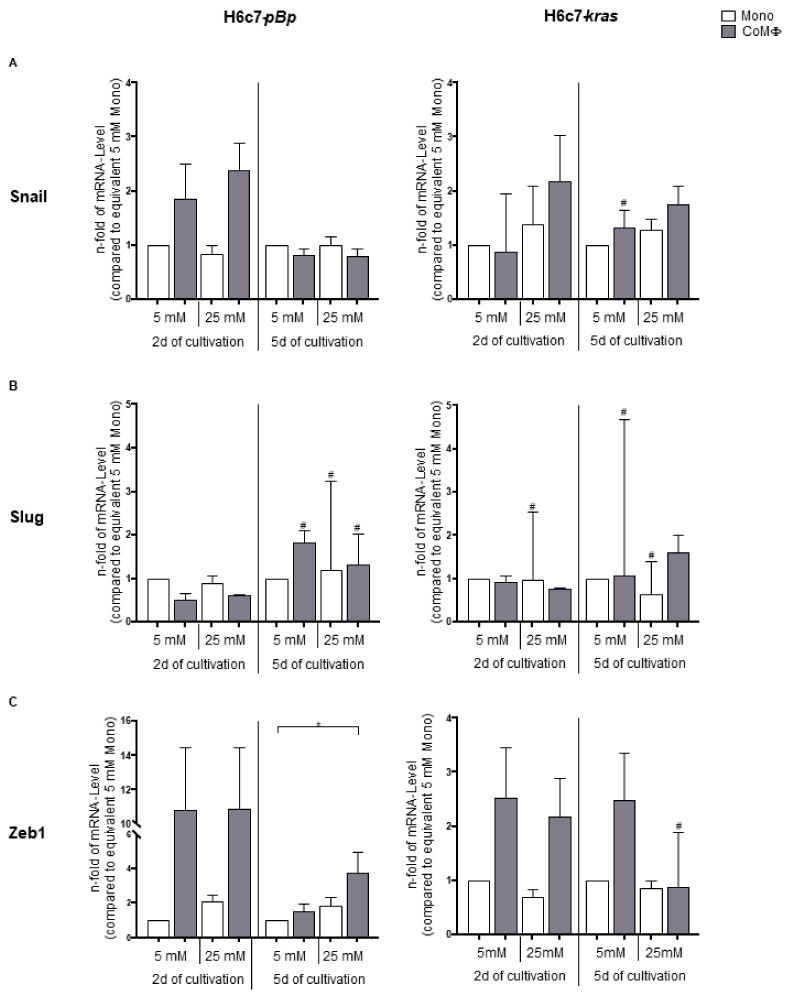
Exposure to M1 macrophages and hyperglycemia alters the mRNA levels of EMT- and CSC-associated transcription factors in PDEC. H6c7-*pBp* or H6c7-*kras* cells were cultivated in mono- or coculture with M1-polarized macrophages (MΦ) under normo- or hyperglycemic conditions (5 or 25 mM of d-glucose) for 2 or 5 days. The epithelial cells were separated from direct coculture with macrophages via CD11b-MACS depletion of M1-MΦ or harvested from monoculture and used for qRT-PCR analysis. The relative mRNA levels of Snail (**A**), Slug (**B**) and Zeb1 (**C**) for both cell lines are depicted. They are normalized to the housekeeping gene GAPDH and presented as *n*-fold expression compared with the monocultured 5 mM sample from the equivalent cultivation timespan. Normally distributed data are presented as mean and standard error of mean; not normally distributed data (indicated via #) are shown as median and interquartile range. * 0.05 > *p* > 0.0332; *n* = 4 for 2-day culture; *n* = 7 for 5-day culture.

Next, we investigated how the presence of M1 macrophages, hyperglycemia or a combination of both impact mRNA levels of EMT and CSC transcription factors in PDEC. Analysis of basal mRNA levels under normoglycemic monoculture revealed that mRNA levels of Snail and Zeb1 were higher in H6c7-*kras* cells, whereas Slug mRNA levels were higher in H6c7-*pBp* cells (Figure S2). While Snail expression was upregulated in benign H6c7-*pBp* cells in a coculture-dependent manner after 2 days (1.80-fold and 2.37-fold elevation through normo- and hyperglycemic coculture, respectively, compared with normoglycemic monoculture), this upregulation did not remain stable over 5 days of culture. In the premalignant H6c7-*kras* cells, exposure to both hyperglycemia and macrophages led to an increase in mRNA levels of Snail at both times of analysis. Hence, the highest mRNA levels of Snail were detected in hyperglycemic coculture, which were elevated 2.17-fold and 1.75-fold compared with 2- and 5-day normoglycemic monoculture, respectively (Figure 2A).

In H6c7-*pBp* cells, Slug mRNA levels were mostly enhanced by 5-day coculture with macrophages under normoglycemic conditions, while in H6c7-*kras* cells hyperglycemic coculture with macrophages led to an increased expression of Slug (Figure 2B). Finally, Zeb1 mRNA levels were elevated by coculture with M1 macrophages in both PDEC lines. This induction was most impressive in benign PDEC after short-term exposure to macrophages, where a 10.8-fold coculture-dependent increase was observed, irrespective of the glucose level. While hyperglycemia did not impact Zeb1 mRNA levels in H6c7-*kras* cells at any time of analysis, it led to a clear induction in H6c7-*pBp* cells. Long-term exposure to hyperglycemia and M1 macrophages led to the most pronounced increase in Zeb1 mRNA levels in benign PDEC, leading to a significant 3.79-fold elevation compared with the equivalent normoglycemic monoculture (Figure 2C).

In summary, these data support the view that concomitant exposure to M1 macrophages and hyperglycemia amplifies alterations in the mRNA levels of EMT and CSC transcription factors, most dominantly of Zeb1, in PDEC.

### 2.3. The Presence of M1 Macrophages and Hyperglycemia Changes the Expression of CSC Marker Genes in PDEC but Hardly Impacts on Colony Formation Ability

Having shown that M1 macrophages and hyperglycemia together promote the expression of inducers and transcription factors of an EMT/CSC phenotype in PDEC, we investigated whether CSC properties of these cells were affected, too. 

**Figure 3:**
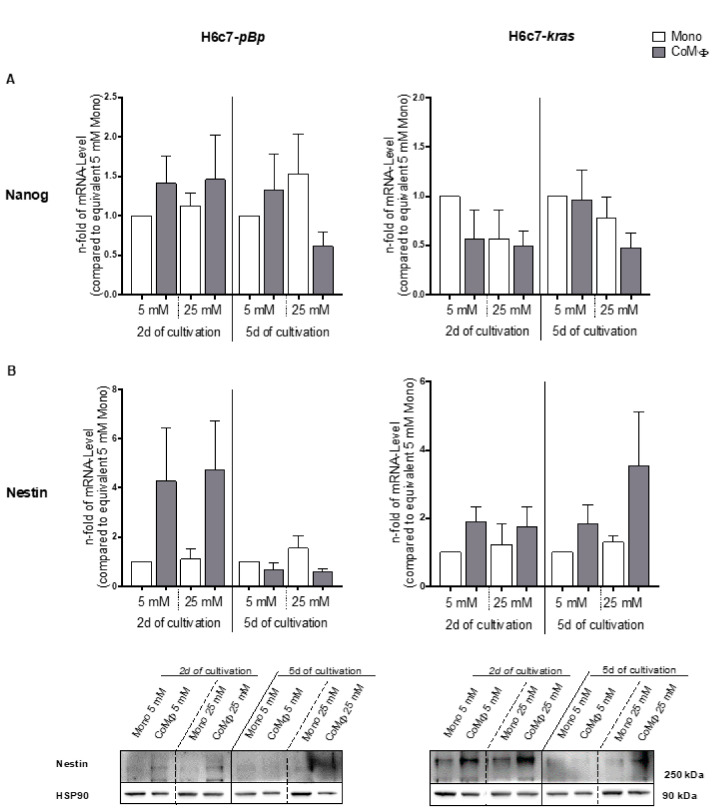
The presence of M1 macrophages and hyperglycemia changes the expression of CSC marker genes in PDEC. H6c7-*pBp* or H6c7-*kras* cells were cultivated in mono- or coculture with M1-polarized macrophages (MΦ) under normo- or hyperglycemic conditions (5 or 25 mM of d-glucose) for 2 or 5 days. The epithelial cells were separated from direct coculture with macrophages via CD11b-MACS depletion of M1-MΦ or harvested from monoculture and used for qRT-PCR analysis. The relative mRNA levels of Nanog (**A**) and Nestin (**B**) for both cell lines are depicted. They are normalized to the housekeeping gene GAPDH and presented as *n*-fold expression compared with the monocultured 5 mM sample from the equivalent cultivation timespan. Normally distributed data are presented as mean and standard error of mean. *n* = 4 for 2-day culture, *n* = 7 for 5-day culture. In (**B**), protein levels of Nestin are also depicted; Hsp90 was used as loading control. A representative of three independent experiments is shown.

As shown in Figure 3A, mRNA levels of the CSC marker Nanog were moderately upregulated by coculture with M1 macrophages in H6c7-*pBp* cells after 2 days. After 5 days of coculture, a similar increase in Nanog mRNA levels could be observed through hyperglycemia and normoglycemic coculture with M1 macrophages, while hyperglycemic coculture with macrophages remarkably had the opposite effect on the benign cells and led to 0.6-fold reduced mRNA levels (Figure 3A). In contrast, in H6c7-*kras* cells, Nanog mRNA levels were reduced by exposure to hyperglycemia or M1 macrophages after 2 days, whereas this reduction could only be observed after hyperglycemic coculture after 5 days (Figure 3A). Although we could observe certain effects on Nanog mRNA levels caused by hyperglycemia and coculture with M1 macrophages, Nanog was not detectable on protein level in either PDEC line under either condition (data not shown), indicating a general low expression of this CSC marker. This was further underlined by the very low basal mRNA levels of Nanog in both PDEC lines under normoglycemic monoculture (Figure S3A).

As seen in Figure 3B, mRNA levels of the CSC marker Nestin were clearly induced by short-term exposure to M1 macrophages and slightly amplified by hyperglycemia. After 5 days, however, hyperglycemia increased, whereas coculture with M1 macrophages reduced Nestin mRNA levels. In contrast, in H6c7-*kras* cells, Nestin mRNA levels were elevated by M1 macrophages and hyperglycemia at both times points. This effect became even more pronounced over time, and the strongest effect could be observed in the premalignant H6c7-*kras* cells when exposed to hyperglycemic coculture for 5 days, reaching a 3.5-fold elevation compared with normoglycemic monoculture and a 1.94-fold increase compared with normoglycemic coculture (Figure 3B). Detection of Nestin was very difficult on protein level (Figure 3B), indicating low expression levels of this CSC marker too, which was further confirmed by low basal Nestin mRNA levels in monocultured PDEC under normoglycemic conditions (Figure S3B). Nevertheless, a coculture-dependent increase in Nestin could be detected in both cell lines after short-term coculture on protein and mRNA level (Figure 3B,C). After 5 days, the strongest effect on protein and mRNA level of Nestin could be observed in premalignant H6c7-*kras* cells exposed to hyperglycemic coculture with M1 macrophages (Figure 3B,C). 

**Figure 4:**
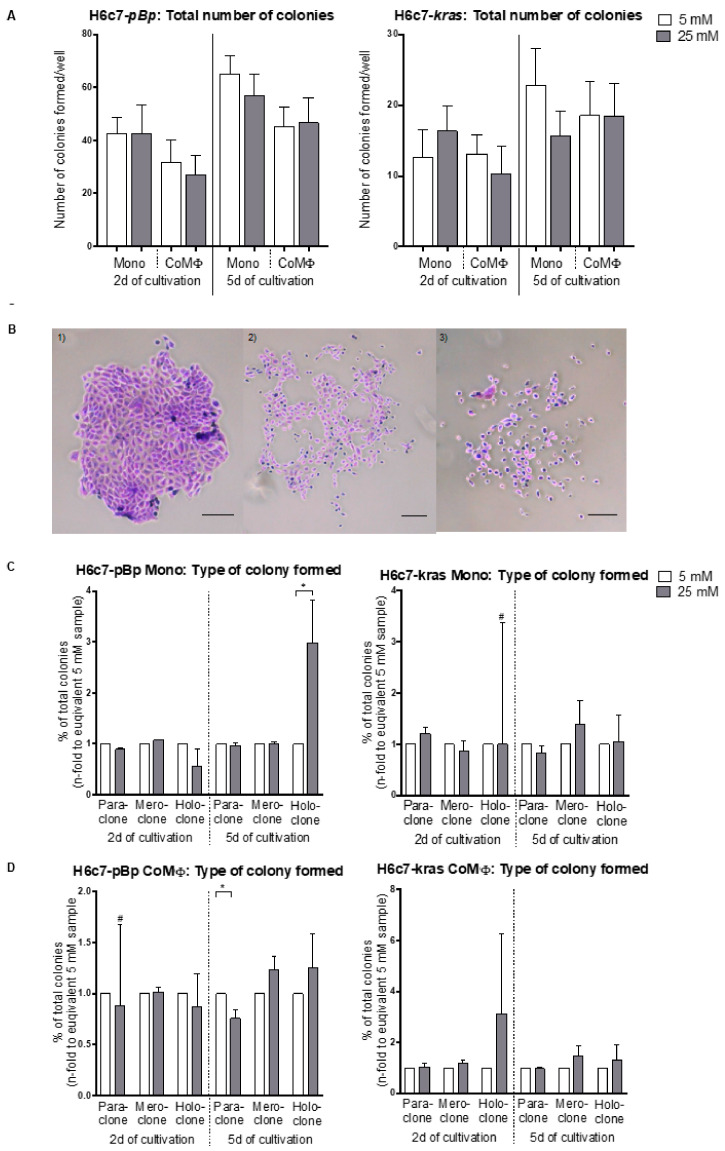
Impact of macrophages and hyperglycemia on the colony formation ability of PDEC. H6c7-*pBp* or H6c7-*kras* cells were cultivated in normo- or hyperglycemic conditions (5 or 25 mM of d-glucose) in mono- or coculture with M1-polarized macrophages (MΦ) for either 2 or 5 days. The epithelial cells were separated from direct coculture with macrophages via CD11b-MACS depletion of M1-MΦ or harvested from monoculture and used for colony formation assays. For this, 800 cells/well were seeded in duplicates and cultivated for further 8 to 14 days. After fixation and staining, colonies of more than 50 cells were analyzed. The total number of colonies per well formed by PDEC arising from short- or long-term mono- or coculture are shown in (**A**). In (**B**) representative pictures of a (1) holo-, (2) mero- and (3) paraclone formed by H6c7-*pBp* cells are shown. The scale bar = 100 µm. The quality of colonies arising from mono- (**C**) or cocultured (**D**) PDEC after short- or long-term cultivation are shown for both cell lines. (**C**,**D**) Data are depicted as *n*-fold to the equivalent sample arising from normoglycemic settings and short-term cultivation. Normally distributed data are presented as mean and standard error of mean; not normally distributed data (indicated via #) are shown as median and interquartile range. * 0.05 > *p* > 0.0332; *n* = 4 for 2-day culture; *n* = 7 for 5-day culture.

Next, we analyzed whether hyperglycemia and macrophage-mediated alterations in CSC marker expression is associated with an altered colony formation ability of PDEC, which is indicative for their self-renewal capacity. Colony formation assays with the differentially cultured PDEC revealed that the total number of colonies formed was hardly affected by coculture with M1 macrophages, hyperglycemia or a combination of both (Figure 4A). However, we could determine a tendency toward formation of more mero- and holoclones, which are thought to contain a higher number of CSC than paraclones [30] in both PDEC lines upon exposure to hyperglycemic conditions, irrespective of whether they had been mono- or cocultured with M1-polarized macrophages (Figure 4C,D).

Overall, these data indicate that PDEC undergo changes in the expression of CSC marker genes (mainly of Nestin) when exposed to M1 macrophages and hyperglycemia but that this does not result in an enhanced self-renewal capacity or elevated colony formation ability.

### 2.4. Combined Impact of M1 Macrophages and Hyperglycemia on the Expression of EMT Marker Genes in PDEC

Next, we analyzed how the presence of M1 macrophages, hyperglycemia or a combination of both impacts the expression of EMT marker genes in PDEC. Analysis of basal expression of E-cadherin, vimentin and L1CAM of monocultured PDEC under normoglycemic conditions demonstrated that H6c7-*kras* cells exhibit a more mesenchymal phenotype than H6c7-*pBp* cells, exemplified by lower mRNA levels of E-cadherin (Figure S4A) and higher mRNA levels of L1CAM and vimentin (Figure S4B,C).

**Figure 5:**
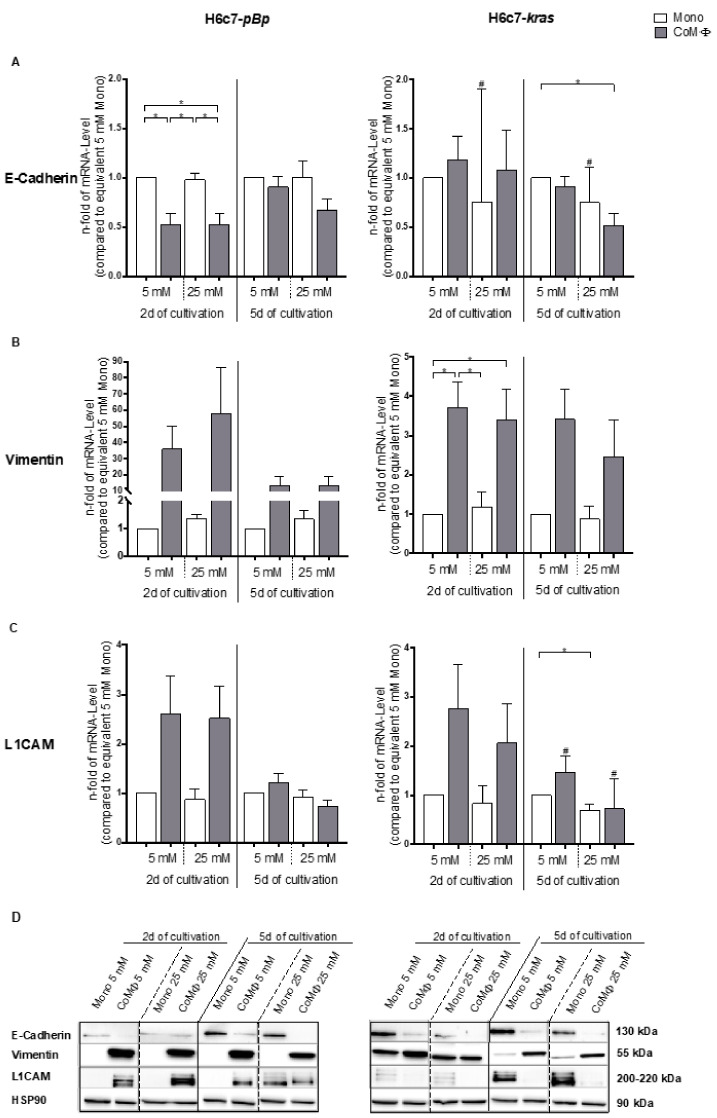
The presence of macrophages and hyperglycemia impacts expression of EMT markers on mRNA and protein level in PDEC. H6c7-*pBp* or H6c7-*kras* cells were cultivated in mono- or coculture with M1-polarized macrophages (MΦ) under normo- or hyperglycemic conditions (5 or 25 mM of d-glucose) for 2 or 5 days. The epithelial cells were separated from direct coculture with macrophages via CD11b-MACS depletion of M1-MΦ or harvested from monoculture and used for qRT-PCR and Western blot analysis. The relative mRNA levels of E-cadherin (**A**), Vimentin (**B**) and L1CAM (**C**) are depicted for both cell lines. They are normalized to the housekeeping gene GAPDH and presented as *n*-fold expression compared to the monocultured 5 mM sample from the equivalent cultivation timespan. Normally distributed data are presented as mean and standard error of mean; not normally distributed data (indicated via #) are shown as median and interquartile range. * 0.05 > *p* > 0.0332; *n* = 4 for 2-day culture; *n* = 7 for 5-day culture. Protein levels of the indicated EMT markers are depicted in (**D**), Hsp90 was used as loading control. A representative of three independent experiments is shown.

As shown in Figure 5A, mRNA levels of the epithelial marker E-cadherin were significantly reduced in H6c7-*pBp* cells after short-term exposure to M1 macrophages, irrespective of the glucose concentration (Figure 5A). In H6c7-*kras* cells, the most pronounced effect of both conditions in combination could be observed on E-cadherin mRNA levels after 5 days, where hyperglycemic coculture led to a significant reduction of 50% compared with normoglycemic monocultured cells.

In contrast, mRNA levels of the mesenchymal marker Vimentin were solely increased by M1 macrophages and almost not affected by hyperglycemia in both PDEC lines (Figure 5B). Interestingly, these effects were more pronounced after 2 than after 5 days and considerably less distinct in premalignant than in benign PDEC. Whereas the most prominent increase (57-fold) could be observed in H6c7-*pBp* cells after hyperglycemic 2-day coculture with macrophages, 2-day exposure to macrophages under either glucose concentration led to a significant elevation in Vimentin mRNA levels in H6c7-*kras* cells. Expression of L1CAM, which has been associated with an EMT and CSC phenotype [31,32], was clearly elevated on mRNA level in both PDEC lines by M1 macrophages under either glucose condition (Figure 5C). Remarkably, this effect was predominantly observed after 2-day coculture. Moreover, alterations in the expression of EMT markers could also be determined on protein level, which further underscore the combined effect of M1 macrophages and hyperglycemia on the reduction of E-cadherin expression (Figure 5D). 

Altogether, these data indicate a combined EMT-promoting effect in PDEC by concomitant exposure to M1 macrophages and hyperglycemia.

### 2.5. Combined Impact of M1 Macrophages and Hyperglycemia on the Migratory Ability of PDEC

**Figure 6:**
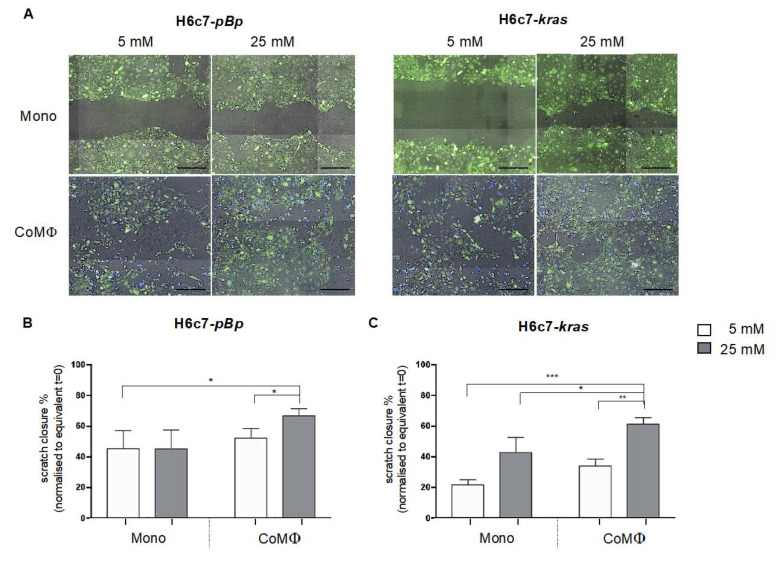
Exposure to macrophages and hyperglycemia promotes the migratory capacity of PDEC. H6c7-*pBp* and H6c7-*kras* cells were stained with CFSE and either mono- or cocultured with CellTrace^TM^ Violet stained M1-polarized macrophages (MΦ) for 2 days in normo- or hyperglycemic settings (5 or 25 mM of d-glucose). The migratory potential was evaluated via scratch assay using the NyONE Cell Imager. Scratch closure was assessed at t = 0 h and t = 16 h for H6c7-*pBp* cells (**A** and **B**) and at t = 0 h and t = 8 h for H6c7-*kras* cells (**A** and **C**). In (**A**), representative images are shown at t = 16h for H6c7-*pBp* cells and at t = 8 h for H6c7-*kras* cells taken with the NyONE imager. The scale bar=1000 µm. In (**B** and **C**), data are expressed as % scratch closure normalized to t = 0 h. Normally distributed data are presented as mean and standard error of mean; * *p* < 0.05; ** = *p* < 0.01; *** = *p* < 0.001; *n* = 3.

To analyze whether the macrophage and hyperglycemia mediated EMT-associated alterations result in a higher migratory ability of PDEC, cell migration of either PDEC line was analyzed by scratch assay after the different culture conditions. Cell migration of H6c7-*pBp* cells was slightly increased after coculture with M1 macrophages under normoglycemic conditions but significantly elevated upon concomitant exposure to macrophages and high glucose levels (Figure 6A,B). Similarly, the strongest and significant migration-promoting effect was observed in H6c7-*kras* cells after simultaneous exposure to both M1 macrophages and hyperglycemia. However, an increase in the migratory capacity could also be observed when H6c7-*kras* cells were exposed to either hyperglycemia or macrophages alone. (Figure 6A,C). Overall, these data underscore that exposure to M1 macrophages and hyperglycemia promotes an EMT phenotype along with enhanced migratory abilities in PDEC.

### 2.6. Blockade of IL-6 by Tocilizumab Increases Both Expression of Epithelial and Mesenchymal Markers in Cocultured Hyperglycemic H6c7-kras Cells and Reduces Cell Migration

**Figure 7:**
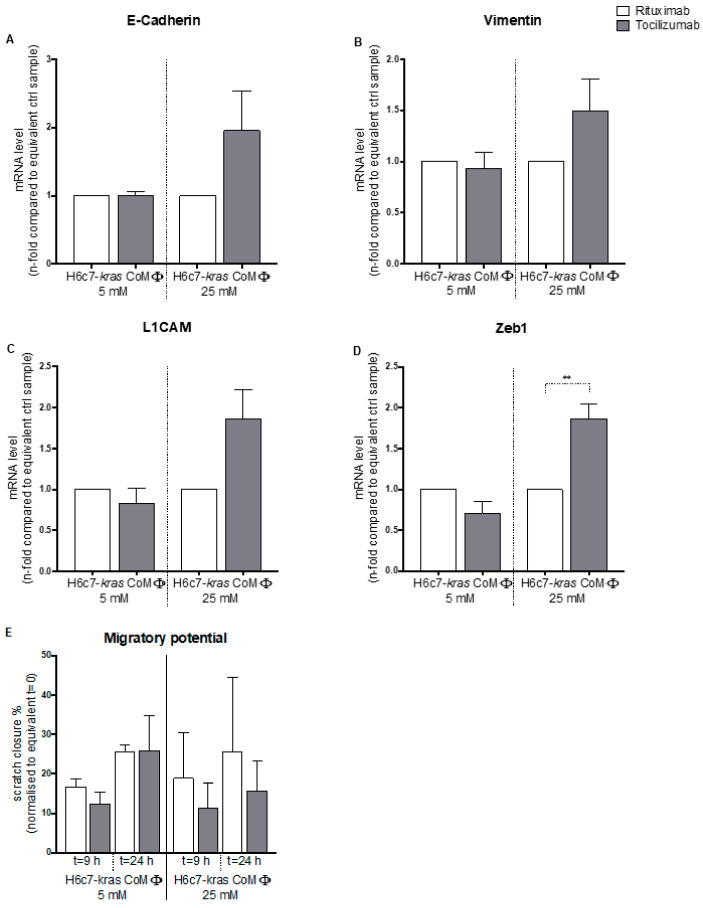
Blockade of IL-6 activity via Tocilizumab increases mRNA levels of epithelial and mesenchymal markers in cocultured hyperglycemic H6c7-*kras* cells and reduces cell migration. H6c7-*kras* cells were cocultured with M1-polarized macrophages (MΦ) for 5 days in normo- or hyperglycemic settings (5 or 25 mM of d-glucose) and treated with 1 μg/mL of Tocilizumab or Rituximab. The epithelial cells were separated from direct coculture via CD11b-MACS depletion of M1-MΦ and used for qPCR analysis. The relative mRNA levels of E-cadherin (**A**), Vimentin (**B**), L1CAM (**C**) and Zeb1 (**D**) are depicted, normalized to the housekeeping gene GAPDH and presented as *n*-fold expression compared with the equivalent control sample. In (**E**), the migratory potential of differentially cultured and treated H6c7-*kras* cells is shown. The migratory potential was monitored via scratch assay using the Lionheart FX Automated Microscope. Scratch closure was assessed at t = 0 h, t = 9 h and t = 24 h and data are expressed as % gap closure normalized to t = 0 h. Normally distributed data are presented as mean and standard error of mean. ** 0.0331 > *p* > 0.0021; *n* = 4.

Because IL-6 mRNA levels were enhanced in H6c7-*kras* cells by the presence of M1 macrophages under normo- and hyperglycemic conditions after 5 day culture (Figure 1) and were also observed to be most impressively elevated in macrophages that had been exposed to PDEC and/or high glucose levels (Figure S5A), we neutralized IL-6 activity by application of Tocilizumab or treated the cells with the control vehicle Rituximab under the different culture conditions to investigate whether IL-6 is involved in the observed EMT-associated alterations. As seen in Figure 7A, treatment with Tocilizumab but not Rituximab clearly prevented the decreasing effect of the 5-day hyperglycemic coculture with M1-polarized macrophages on E-cadherin mRNA levels in H6c7-*kras* cells (1.96-fold elevation in E-cadherin mRNA levels through Tocilizumab treatment compared with the equivalent cells treated with Rituximab). However, a concomitant induction of mRNA levels of mesenchymal Vimentin (Figure 7B), L1CAM (Figure 7C) and Zeb 1 (Figure 7D) was also noted, suggesting that the EMT phenotype was only partially reverted by blocking IL-6 activity. Importantly, in line with the increased E-cadherin mRNA levels, Tocilizumab-treated H6c7-*kras* cells that had been exposed to M1 macrophages and hyperglycemia showed a reduced migratory ability in the scratch assay compared with the respective cells treated with Rituximab (Figure 7E). 

Whereas only 15.61% of the scratch were closed by the equivalent cells treated with Tocilizumab, control cells showed a scratch closure of 25.68% after 24 h.

These data underline the important role of E-cadherin reduction in conferring a migratory phenotype to premalignant PDEC and suggest IL-6 to play a role in the acquisition of an EMT phenotype of H6c7-*kras* cells provoked by hyperglycemia and M1 macrophages.

### 2.7. Neutralization of TNF-α Reverses the Effects of M1 Macrophages and Hyperglycemia on the EMT Phenotype in H6c7-Kras Cells

Having shown that TNF-α mRNA levels were significantly elevated in H6c7-*kras* cells after long-term and in H6c7-*pBp* cells after short-term exposure to normo- and hyperglycemic coculture with M1 macrophages conferring an EMT phenotype in these cells, we neutralized the effect of TNF-α by application of Etanercept (ETN). Untreated cells from the different culture conditions served as a control. 

**Figure 8:**
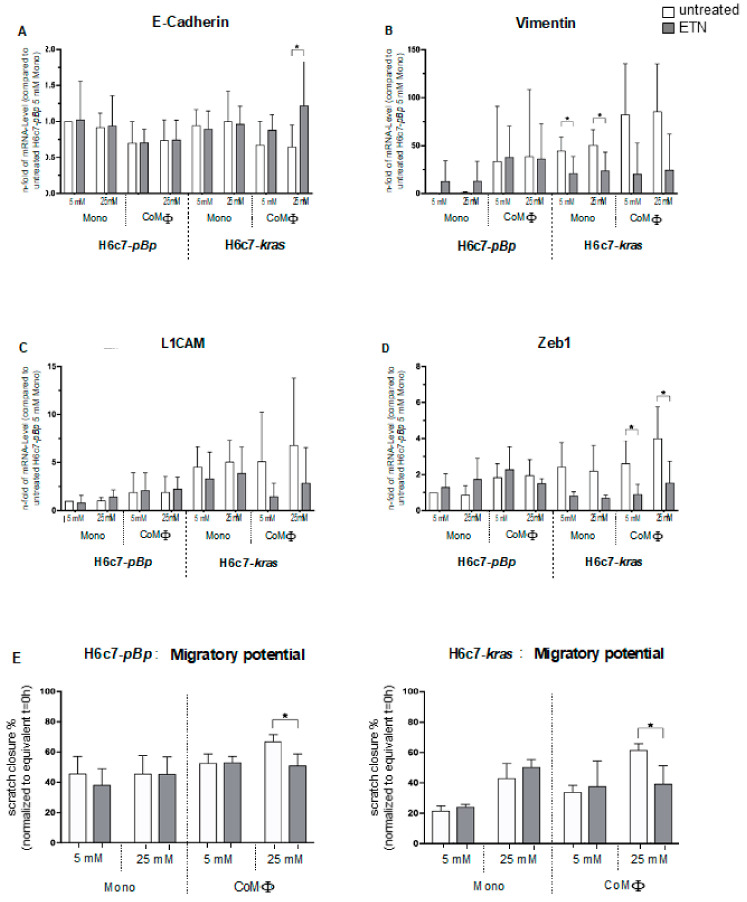
Neutralization of TNF-α reverses the effects of M1 macrophages and hyperglycemia on the EMT phenotype in H6c7-*kras* cells. CFSE-stained H6c7-*pBp* or H6c7-*kras* cells were cultivated in mono- or coculture with M1-polarized macrophages (MΦ) under normo- or hyperglycemic conditions (5 or 25 mM of d-glucose). Blockade of TNF-α activity was achieved by treatment with 10 μM Etanercept (ETN). As control, cells were left untreated. The epithelial cells were separated from direct coculture via CD11b-MACS depletion of M1-MΦ and used for qPCR analysis. The relative mRNA levels of E-cadherin (**A**), Vimentin (**B**), L1CAM (**C**) and Zeb1 (**D**) are depicted, normalized to the housekeeping gene GAPDH and presented as *n*-fold expression compared with the respective population of monocultured H6c7-*pBp* cells in 5 mM glucose medium without ETN. Normally distributed data are presented as mean and standard error of mean; *n* = 9 ⌀ ETN; *n* = 3 +ETN. In (**E**) the migratory potential of PDEC mono- or cocultured with M1-MΦ under both glucose conditions with or without ETN treatment is shown. For this, PDEC were stained with CFSE and macrophages with CellTrace^TM^ Violet, and a scratch assay was performed using the NyONE Cell Imager. Scratch closure was recorded at t = 0 h and t = 16 h for H6c7-*pBp* cells as well as t = 0 h and t = 8 h for H6c7-*kras* cells and data are presented as % scratch closure. Normally distributed data are presented as mean and standard error of mean; * *p* < 0.05; *n* = 3.

In the benign H6c7-*pBp* cells, mRNA levels of neither epithelial E-cadherin (Figure 8A) nor the mesenchymal markers L1CAM and Zeb1 were impacted by ETN treatment (Figure 8 C,D). However, mRNA levels of the mesenchymal marker Vimentin were elevated in monocultured H6c7-*pBp* cells treated with ETN (Figure 8B).

In premalignant H6c7-*kras* cells, the effects of ETN treatment were more pronounced. In line with our previous findings, we observed the strongest effect of ETN on H6c7-*kras* cells cocultured under hyperglycemic conditions, leading to a significant induction of E-cadherin mRNA levels (Figure 8A). Furthermore, decreased mRNA levels of the mesenchymal markers Vimentin (Figure 8B), L1CAM (Figure 8C) and Zeb1 (Figure 8D) were observed in ETN-treated cells under all conditions compared with corresponding untreated cells.

While ETN treatment did not alter cell migration of either benign or premalignant PDEC after normo- or hyperglycemic monoculture nor in normoglycemic coculture with M1-polarized macrophages, a significant reduction of cell migration could be observed in both PDEC lines after hyperglycemic coculture (Figure 8E). These findings are in line with the clearly elevated E-cadherin expression and reduced expression of mesenchymal markers in H6c7-*kras* cells treated with ETN under hyperglycemic coculture. These findings support the view that hyperglycemia and M1 macrophages promote a TNF-α-dependent migratory EMT phenotype in PDEC.

## 3. Discussion

Compelling evidence already exists that tumorigenesis in PDAC is promoted by macrophages [20] or hyperglycemia [33], but little is known about how they may impact PDAC initiation and progression concomitantly. However, macrophages as well as elevated glucose levels are closely linked, as both are crucial mediators of an inflammatory milieu. Regarding the alarming increase in the incidence of metabolic disorders such as T2DM and obesity [34,35,36]—which are characterized by such hyperglycemic inflammatory tissues—the understanding of underlying molecular mechanisms in promotion of tumorigenesis is of great importance. Therefore, our study aimed at elucidating the mechanisms by which a pro-inflammatory and hyperglycemic surrounding promotes the acquisition of malignancy-associated alterations in benign and premalignant PDEC.

In our study, a glucose- and macrophage-dependent EMT induction was observed in both H6c7-*pBp* and H6c7-*kras* cells used as model for benign and premalignant PDEC, respectively. Loss in E-cadherin expression—commonly referred to as a milestone in EMT—was already observed after 2 days of coculture in benign PDEC, whereas the most prominent impact was seen upon concomitant long-term exposure to macrophages and high glucose levels in H6c7-*kras* cells. In line with the finding that Snail represses E-cadherin and other epithelial molecules [37], Snail mRNA levels were most strongly elevated after short-term exposure to macrophages and hyperglycemia in H6c7-pBp cells and were increased after short- but also after long-term exposure in H6c7-*kras* cells. Supporting this observation, several other studies demonstrated that the expression of Zeb1, Slug and Snail is closely intertwined with the repression of E-cadherin levels, and enhanced level of either transcription factor thus promotes EMT [38,39]. The fact that reduction of E-cadherin expression after long-term culture was more pronounced in the premalignant than in the benign PDEC implies a dependency on the *Kras* mutation. Coinciding with this hypothesis, several mouse models demonstrated that caerulein-induced pancreatic inflammation leads to tumor progression exclusively in mice harboring *Kras* mutation [40,41]. As a possible explanation for this phenomenon, Daniluk et al. suggest the idea of an inflammation-induced positive feedback loop via NF-κB that is strictly dependent on oncogenic *Kras* causing an amplification of kras levels up to pathological levels, thereby promoting tumorigenesis [42]. Moreover, Storz et al. identified the *Kras* mutation as a mediator of ICAM-1 upregulation that attracts M1-polarized macrophages and promotes secretion of pro-inflammatory cytokines and proteases, also leading to tumor progression [43]. In this context, we observed that M1-polarized macrophages are pushed toward an even more pro-inflammatory phenotype by the presence of high glucose levels and PDEC, especially H6c7-*kras* cells (Figure S1). Altogether, this might also explain the pronounced effect in H6c7-*kras* cells after long-term exposure to an inflammatory hyperglycemic microenvironment. However, other mechanisms by which inflammatory processes promote EMT also seem to be important, as e.g., the upregulation of Vimentin and Zeb1 mRNA levels was more prominent in H6c7-*pBp* than in H6c7-*kras* cells, even though the basal mRNA levels of these markers were higher in the premalignant cells (Figures S2 and S4). In line with these findings, the migratory potential of both PDEC lines was elevated through concomitant exposure to macrophages and high glucose levels. However, scratch closure of H6c7-*pBp* cells was 2-fold slower compared with H6c7-*kras* cells, supporting the view that H6c7-*kras* cells already exhibit a more pronounced basal migratory potential that is in line with the higher basal expression levels of mesenchymal markers. Altogether, these findings indicate that an inflammatory hyperglycemic microenvironment already impacts the acquisition of malignancy-associated alterations in very early stages of pancreatic tumorigenesis.

IL-6 is a pro-inflammatory cytokine known to be elevated in T2DM patients, and it is even attributed a prognostic value concerning the risk of T2DM onset [44,45]. Similarly, elevated IL-6 serum levels were shown to be associated with a poorer prognosis in various cancer entities [46,47,48]. As IL-6 mRNA levels were elevated in H6c7-*kras* cells exposed to macrophages irrespective of the glucose concentration and were strongly modulated in macrophages upon concomitant exposure to PDEC and glucose (Figure S1A), IL-6 activity was blocked to determine whether this cytokine is a mediator of the EMT-associated alterations in PDEC. The blockade of IL-6 activity via Tocilizumab (impairing classic and trans-signaling) predominantly induced EMT characteristics in H6c7-*kras* cells from hyperglycemic conditions indicated by increased mRNA levels of the mesenchymal markers Vimentin, L1CAM and Zeb1. However, the epithelial E-cadherin was also upregulated and the migratory capacity reduced, indicating a partial EMT reversion. Our findings partly stand in contrast to observations of Wan et al., who attributed great importance to elevated TAM-derived IL-6 levels in the context of invasiveness and acquisition of stemness features of hepatocellular carcinoma cell lines. They showed a reversion of these effects in vitro and reduced tumor growth in a xenograft mouse model upon Tocilizumab treatment [49]. However, Mauer et al. showed that the expression levels of genes linked to inflammatory processes and insulin resistance were significantly higher in liver and white adipose tissue of mice with an IL-6 deficiency in myeloid cells than in mice with intact IL-6 activity [50], which is consistent with our observations that in cocultured H6c7-*kras* cells mRNA levels of EMT- and CSC-inducing pro-inflammatory mediators were elevated upon Tocilizumab treatment (data not shown). Furthermore, the group revealed that the interruption of IL-6 activity leads to a greater number of M1-polarized macrophages in mice fed with a high-fat diet compared with mice with a normal diet and intact IL-6 activity, which further underlines the gain in pro-inflammatory properties [50].

Overall, the current findings revealed that IL-6 is indeed closely intertwined with the acquisition of malignancy-associated alterations in premalignant PDEC. This is further supported by the study of Lesina et al., which demonstrated that the progression of PanIN to PDAC is driven by *KrasG12D* mutations as well as the interplay of PDEC and myeloid cells, the latter being highly dependent on IL-6 [51].

The pro-inflammatory cytokine TNF-α has been associated with the acquisition of both EMT and CSC characteristics in various tumor entities, including PDAC [46,52,53,54,55]. In the current study, TNF-α mRNA levels peaked in a macrophage-dependent manner in the benign PDEC after 2 days and decreased again after 5 days. In the premalignant PDEC, on the other hand, the concomitant exposure to a pro-inflammatory and hyperglycemic microenvironment elevated TNF-α levels over time. This could be confirmed by multiplex assay revealing the highest TNF-α levels in supernatants of cocultured H6c7-*kras* cells under hyperglycemic conditions (Figure S6). With respect to CSC markers, Nestin mRNA levels were similarly altered in both cell lines, suggesting a link here. This is corroborated by previous findings of our group that could show that blockade of TNF-α in holoclones of the PDAC cell line Panc1 reduces CSC properties, including Nestin expression [56]. The differing impact of glucose and macrophages on TNF-α levels in the two PDEC cell lines could be explained by the differential contribution of macrophages and their respective cytokine expression profile: TNF-α mRNA levels in M1-polarized macrophages were elevated even more explicitly in the presence of H6c7-*kras* than of H6c7-*pBp* cells (Figure S5C). In line with this finding, Bates et al. suggested a link between macrophages’ TNF-α expression and a thereby induced autocrine TNF-α production by epithelial cells that is dependent on ERK activation [57]. This could explain the stronger increase of TNF-α mRNA levels in premalignant than in benign PDEC after 5 days and further corroborates our findings of a complex crosstalk between macrophages and epithelial cells promoting and self-amplifying a favorable inflammatory microenvironment for the acquisition of EMT- and CSC-associated properties in PDEC. Furthermore, Csiszar et al. could correlate cancer progression to rising TNF-α levels as tumor cells abundantly release TNF-α compared with benign epithelial cells of the colon [58]. Notably, these findings coincide with those of our current study as the basal expression level of TNF-α was considerably lower in H6c7-*pBp* than in H6c7-*kras* cells, suggesting a role of TNF-α in EMT induction in premalignant and malignant PDEC. This is further supported by the fact that blockade of TNF-α via ETN led to a reversal of the EMT phenotype in the premalignant but not in benign PDEC. In H6c7-*kras* cells, a clear induction of the epithelial marker E-cadherin and a loss in mesenchymal markers could be observed upon ETN treatment. However, the migratory potential of both PDEC lines was reduced upon ETN treatment, indicating that TNF-α promotes a migratory phenotype in benign PDEC by other mechanisms than EMT induction. However, in premalignant PDEC, TNF-α seems to enhance migratory abilities via induction of EMT. This is in line with the findings of Wang et al. who showed that diabetic culture conditions promote proliferation and invasiveness of pancreatic cancer cells via elevated TNF-α and NF-κB levels [33]. Inhibition of this signaling cascade decreased tumor size and ameliorated survival rates in diabetic mice bearing pancreatic tumors.

Altogether, this study provides a first hint with regard to which mediators contribute to pancreatic malignant progression driven by metabolic disorders. Blockade of IL-6 signaling or TNF-α has already been shown to be effective in reducing tumor growth and metastasis in preclinical studies using PDAC mouse models as well as in the therapy of other tumor entities and inflammatory diseases [52,59,60,61,62,63,64]. An inflammatory milieu already manifests in early precancerous lesions and is further promoted by hyperglycemia via recruitment of macrophages as well as self-amplifying pro-inflammatory signaling cascades. This may call for future studies that investigate blocking of either factor at early stages in pancreatic tumorigenesis and might therefore be a possible supplementary point of attack that undermines malignant progression in PDAC.

## 4. Materials and Methods

### 4.1. Cell lines

H6c7-*pBp* and H6c7-*kras* cells that represent a benign and a premalignant phenotype of PDEC, respectively, originated from the non-tumorigenic human pancreatic ductal epithelial (HPDE) cell line and were immortalized by transduction with the HPV-16 E6/E7 oncogenes [65,66]. Cell lines were generated and donated by M.S. Tsao (Ontario Cancer Institute, Toronto, Canada). Cells were cultured in 8 mM d-glucose H6c7 medium composed of 50% RPMI 1640 medium (Biochrom, Berlin, Germany) and 50% keratinocyte serum-free (KSF) medium (Gibco Life Technologies, Darmstadt, Germany) supplemented with 5% fetal calf serum (FCS), 1 mM l-glutamine (both Biochrom, Berlin, Germany), 25 mg/mL bovine pituitary extract, 2.5 ng/mL epidermal growth factor (both Gibco Life Technologies, Darmstadt, Germany) and 0.5 mg/mL puromycin (Invitrogen, Darmstadt, Germany). Cells were cultured at 37 °C, 5% CO_2_ and 85% humidity.

### 4.2. Generation of Macrophages

To generate in vitro M1-polarized macrophages, monocytes were isolated from blood of healthy donors, who had given their informed consent. Approval was obtained by the ethics committee of the Medical Faculty at Kiel University (reference number: D490/17, approved on 23 June 2017). As byproducts of platelet donation, leukoreduction system chambers (LRSCs) provide a source of white blood cells including monocytes. As the LRSC contained concentrated leukocytes, peripheral blood mononuclear cells (PBMCs) first had to be separated from other blood components such as red blood cells, granulocytes and debris. Isolation of monocytes by counterflow centrifugation as well as the differentiation cultures for macrophages were performed as described [20,67]. The phenotype of macrophages was characterized following established protocols [68,69,70].

### 4.3. Direct Coculture of PDEC and Macrophages

To approximate the proportion of epithelial cells and macrophages during pancreatic tumorigenesis, a cell number of 5 × 10^4^ PDEC/well was chosen for 2-day coculture. For long-term (5-day) coculture, 2 × 10^4^ per well were chosen for the benign H6c7-*pBp* cells, whereas 3 × 10^4^ premalignant H6c7-*kras* cells were seeded per well considering the different cell division rates of PDEC. PDEC were seeded into a 12-well plate one day prior to seeding of macrophages in 1 mL of pre-warmed medium to assure their proper attachment. In initial experiments (and as indicated in the figure legends), H6c7-*pBp* or H6c7-*kras* cells were stained with CFSE (Thermo Scientific, Schwerte, Germany, used in a dilution of 1:1000) prior to their seeding to allow flow cytometric analysis of the purity of PDEC once they were separated from macrophages at the end of coculture. Once an adequate purity was ensured, subsequent coculture experiments were performed without CFSE staining of PDEC. Irrespective of CFSE labelling of PDEC, M1-polarized macrophages were harvested as described [70] and resuspended in the respective, pre-warmed coculture medium (RPMI 1640 medium (without any glucose)) supplemented with 10% FCS, 2 mM l-glutamine and either 5 mM or 25 mM d-glucose, the latter concentration being commonly used to model hyperglycemic conditions [71,72]. An amount of 2.5 × 10^5^ macrophages/well was seeded directly onto PDEC the next day. The medium of monocultured cells was aspirated as well and replaced by 1 mL of the respective coculture medium. Mono- and cocultures were maintained for 2 or 5 days at 37 °C, 5% CO2 and 85% humidity.

### 4.4. Blocking of IL-6 and TNF-α

To analyze the potential contribution of TNF-α and IL-6 on the acquisition of EMT-related properties of PDEC under coculture with macrophages and under hyperglycemic stress, blockade experiments were performed. For the blockade of TNF-α, 10 µM Eternacept (ETN, Pfizer, Berlin, Germany) was applicated on day 1 of normo- or hyperglycemic coculture. Untreated cocultured H6c7-*kras* cells in the equivalent conditions served as a control. 

IL-6 activity was blocked by adding 1 µg/mL of Tocilizumab (Hoffmann La Roche, Basel, Switzerland). As control, Rituximab (kindly provided by Prof. Dr. Matthias Peipp, Kiel) also being a human IgG1 antibody but targeting the B cell-specific CD20 protein not being present in our culture system, was added at the same concentration to H6c7-*kras* cells on day 2 of coculture. 

### 4.5. Magnetic Activated Cell Sorting

To analyze the effect of macrophages on PDEC, H6c7-*pBp* or H6c7-*kras* cells were separated after 2- or 5-day coculture via magnetic activated cell sorting (MACS). Separation of H6c7-*pBp* and H6c7-*kras* cells from macrophages was achieved by depletion of the macrophage population via targeting the surface molecule CD11b. For this purpose, supernatants were aspirated, and cells were detached with Accutase (EMD Millipore Corporation, Temecula, CA, USA). Once they were suspended, all cells exposed to the same treatment were collected in 50 mL tubes and centrifuged for 10 min at 300× *g* before they were resuspended in 3 mL ice-cold MACS buffer (0.5% BSA in PBS supplemented with 2 mM EDTA). Then, cell suspensions were pipetted onto a Pre-Separation Filter (Milteny Biotec GmbH, Bergisch Gladbach, Germany), which had been moisturized with 500 μL MACS buffer. The filter was then washed with 500 μL MACS buffer thrice and the filtered suspensions again centrifuged for 10 min at 300× *g*. Afterward, cells were resuspended in 80 μL MACS buffer + 20 μL of CD11b microbeads (Milteny Biotec GmbH, Bergisch Gladbach, Germany), thoroughly mixed and incubated for 15 min at 4 °C. Then, the cell suspension was washed with 2 mL MACS buffer and centrifuged for 10 min at 300× *g*. During centrifugation, LD-columns (Milteny Biotec GmbH, Bergisch Gladbach, Germany) were placed into the magnet and primed with 2 mL MACS buffer. The cells were resuspended in 500 μL MACS buffer and the cell suspension transferred into the columns to start the separation. The flow through containing PDEC was collected in new 50 mL tubes. To retrieve remaining PDEC from the column, it was washed three times with 1 mL MACS buffer. Finally, the collected cells were counted and used in subsequent experimental settings. The purity of PDEC after MACS-mediated depletion of macrophages was 94–98% as determined via flow cytometry. Additionally, the macrophage marker CD163 could hardly be detected in PDEC arising from coculture with M1-polarized macrophages but in M1 macrophage monoculture (data not shown).

### 4.6. Colony Formation Assay

Colony formation assays (CFA) were performed to assess the self-renewal capacity of differentially cultured PDEC. For seeding of CFA, cells were detached, and 400 single cells were seeded into a 6-well plate as duplicates in 5 mM or 25 mM d-glucose containing coculture media. After cultivation for 8 to 10 days, colonies were washed in PBS and fixed with 4.5% PFA for 15 min at RT. Then, colonies were stained with 0.1% crystal violet for 1 h at RT, washed in water and air-dried overnight. Only colonies with more than 50 cells were considered as colony and counted. Additionally, the morphology of each colony was assessed and assigned as para-, mero- or holoclone [73,74].

### 4.7. Scratch Assay

To analyze cell migration of differentially cultured PDEC, a scratch assay was performed. For this purpose, the cells were seeded as described above; PDEC were stained with CFSE and macrophages with CellTrace^TM^ Violet (Thermo Scientific, Schwerte, Germany) in order to discriminate both cell types. After 2 days of mono- or coculture, a straight scratch was applied in the middle of each well. Afterward, the medium was discarded and replaced by 1 mL of prewarmed scratch assay medium containing only 1% FCS, ensuring that gap closure was not facilitated by cell growth and could therefore be attributed to migration of the cell layers. Right after applying the scratch, the wells were photographed by the NyONE Cell Imager (Synentec GmbH, Elmshorn, Germany) (t = 0 h). Next, pictures of mono- and cocultured H6c7-*pBp* and H6c7-*kras* cells were taken after 8 and 16 h, respectively, due to different migration rates of the two PDEC types.

For analysis of the migratory potential of cocultured PDEC after tocilizumab treatment, cells were seeded as described above and treated with 1 µg/mL of tocilizumab or rituximab as control on day two of coculture. On day five, a scratch was performed as described above, and the cells were immediately photographed with the Lionheart FX Automated Microscope (BioTek, Bad Friedrichshall, Germany) (t= 0 h). Further pictures were taken 9 and 24 h after the scratch had been applied.

For analysis of wound closure, the wound surfaces at the different timepoints were compared with the equivalent surface at t = 0 h using the Gen5 Data Analysis Software (BioTeK, Bad Friedrichshall, Germany) or the ImageJ software (Wayne Rasband, National Institutes of Health, Bethesda, MA, USA).

### 4.8. Multiplex Assay for Determination of TNF-α in Culture Supernatants

Quantification of TNF-α concentration in culture supernatants was performed with the LEGENDplex Human Inflammation Panel 1 Multi-Analyte Flow Assay Kit (Biolegend, Koblenz, Germany) according to the manufacturer’s instructions. For sample preparation, supernatants of respective culture conditions were centrifuged for 30 min at 15,000× *g* and 4 °C. Afterward, supernatants were separated and stored at –80 °C multiplex until assay procedure. Detection of assay beads was performed with a FACSVerse flow cytometer and FACSDiva software (Becton Dickinson, Heidelberg, Germany). Multiplex evaluation was executed with the included LEGENDplex software v8.0 (Biolegend).

### 4.9. Western Blot

Preparation of whole cell lysates and nuclear extracts as well as electrophoresis were performed as described [75,76]. The antibodies listed in Table 1 were used according to the manufacturer’s instructions. Primary antibodies were incubated overnight at 4 °C and detected by anti-mouse HRP-linked antibodies (Cell Signalling, Frankfurt, Germany) at room temperature (RT) for 1 h. After washing in TBST, blots were developed with SuperSignal West Dura Extended Duration Substrate (Perbio Sciences, Bonn, Germany).

### 4.10. RNA Isolation and RT-qPCR

Total RNA of differentially cultured PDEC was isolated with the total RNA kit peqGOLD (PeqLab, Erlangen, Germany) and subjected to reversed transcription according to the manufacturer’s instructions (Fermentas, via Thermo Fisher Scientific, Darmstadt, Germany). Primers and primer sequences are listed in Table 2. All PCRs were performed as duplicate analyzed with a LightCycler 480 (Roche, Mannheim, Germany) for 50 cycles, followed by a melting curve analysis. The amount of cDNA of the gene of interest was normalized to the cDNA amount of the control gene GAPDH.

### 4.11. Statistics

Statistical analysis was performed using GraphPad Prism Version 5.00 (GraphPad Software Inc., La Jolla, CA, USA) and SigmaPlot v12.5 (Systat, Erkrath, Germany). Normality was tested by using the Shapiro–Wilk test. Normally distributed data are presented as mean and standard error of mean, and one-way ANOVA or unpaired *t*-test were performed for statistical analysis. Not normally distributed data (indicated via #) are shown as median and interquartile range. Kruskal–Wallis one-way ANOVA on ranks was used for statistical analysis. Results were considered as statistically significant for *p*-values < 0.05. Significance of results is indicated as follows: * 0.05 > *p* > 0.0332; ** 0.0331 > *p* > 0.0021; *** 0.002 > *p* > 0.0001.

## 5. Conclusions

Overall, the current study corroborates the complex interplay of macrophages and hyperglycemia as an important contributor to the acquisition of malignancy-associated alterations in PDEC. A T2DM- and obesity-associated hyperglycemic and inflammatory microenvironment promotes the acquisition of EMT properties in PDEC. Even though these alterations were more evident in the premalignant PDEC already harboring a *Kras* mutation, benign H6c7-*pBp* cells also acquired those malignancy-associated properties in the presence of macrophages and/or hyperglycemia, underscoring the relevance of early prevention options. Since the observed changes could partially be attributed to TNF-α and IL-6 activity, an anti-inflammatory therapy might be a promising early therapeutic prevention option, especially in high-risk patients, such as those suffering from obesity or T2DM.

## Figures and Tables

**Table 1:** Antibodies used for Western Blot.

Antibody	Host	Dilution	Manufacturer and Catalog Number
E-cadherin (clone 32A8)	mouse	1:1000	Cell Signaling, Frankfurt, Germany, #5296S
Vimentin (clone V9)	mouse	1:200	Santa Cruz Biotechnology, Heidelberg, Germany, #SC-6260
HSP90 (clone F8)	mouse	1:2000	Santa Cruz Biotechnology, Heidelberg, Germany, #SC-13119
L1CAM (clone 9.3)	mouse	1:1000	Kindly provided by Prof. Gerd Moldenhauer, Cancer Research Centre, Heidelberg, Germany
Nestin (clone 10C2)	mouse	1:500	Invitrogen, Darmstadt, Germany, #14-9843-82
Mouse-IgG-HPR	horse	1:2000	Cell Signaling, Frankfurt, Germany, #7076

**Table 2:** Primer and Primer Sequences.

Primer	5′-3′-Sequence	Manufacturer
E-cadherin	FW: TGCTCTTGCTGTTTCTTCGG RV: TGCCCCATTCGTTCAAGTAG	RealTime Primers via Biomol, Hamburg, Germany
GAPDH	FW: TCCATGACAACTTTGGTATCGTGG RV: GACGCCTGCTTCACCACCTTCT	Eurofins, Ehrensberg, Germany
IL-6	FW: ATGCAATAACCACCCCTGAC RV: GAGGTGCCCATGCTACATTT	RealTime Primers via Biomol, Hamburg, Germany
IL-8	FW: GTGTGAAGGTGCAGTTTTGCC RV: AACTTCTCCACAACCCTCTGC	RealTime Primers via Biomol, Hamburg, Germany
L1CAM	FW: GAACTGGATGTGGTGGAGAG RV: GAGGGTGGTAGAGGTCTGGT	RealTime Primers via Biomol, Hamburg, Germany
Nestin	FW: GAAACAGCCATAGAGGGCAAA RV: TGGTTTTCCAGAGTCTTCAGTGA	Eurofins, Ehrensberg, Germany
Nanog	FW: ACCTACCTACCCCAGCCTTT RV: CATGCAGGACTGCAGAGATT	RealTime Primers via Biomol, Hamburg, Germany
Slug	FW: ATATTCGGACCCACACATTACCT RV: GCAAATGCTCTGTTGCAGTGA	Biometra, Göttingen, Germany
Snail	FW: CTGCTCCACAAGCACCAAGAGTC RV: CCAGCTGCCCTCCCTCCAC	Biometra, Göttingen, Germany
TGF-β1	FW: CGTGGAGCTGTACCAGAAATA RV: TCCGGTGACATCAAAAGATAA	Eurofins, Ehrensberg, Germany
TNF-α	FV: TCCTTCAGACACCCTCAACC RV: AGGCCCCAGTTTGAATTCTT	Eurofins, Ehrensberg, Germany
Vimentin	FW: TCCAAGTTTGCTGACCTCTC RV: TCAACGGCAAAGTTCTCTTC	RealTime Primers via Biomol, Hamburg, Germany
Zeb1	FW: TCCATGCTTAAGAGCGCTAGCT RV: ACCGTAGTTGAGTAGGTGTATGCCA	Eurofins, Ehrensberg, Germany

## Data Availability

The study does not comprise any publicly archived datasets analyzed or generated.

## Supplementary Materials

The following are available online at https://www.mdpi.com/article/10.3390/ijms22105086/s1.

## Abbreviations

CoMΦ	Coculture with M1-polarized macrophages
CP	Chronic pancreatitis
CSC	Cancer stem cells
EMT	Epithelial–mesenchymal-transition
ETN	Etanercept
GAPDH	Glycerinaldehyd-3-phosphat-dehydrogenase
PanIN	Pancreatic intraepithelial neoplasia
PDAC	Pancreatic ductal adenocarcinoma
PDEC	Pancreatic ductal epithelial cells
T2DM	Type 2 diabetes mellitus
TGF-β1	Transforming growth factor-beta 1
TNF-α	Tumor necrosis factor-alpha
