# Supplementary material for: Initiation of Pancreatic Cancer: The Interplay of Hyperglycemia and Macrophages Promotes the Acquisition of Malignancy-Associated Properties in Pancreatic Ductal Epithelial Cells
Source: Int J Mol Sci. 2021 May 11;22(10):5086. doi: 10.3390/ijms22105086 (PMC8151031; doi:10.3390/ijms22105086)
Supplement: Supplementary file 1 [file ijms-22-05086-s001.zip › Figure S1.pdf]

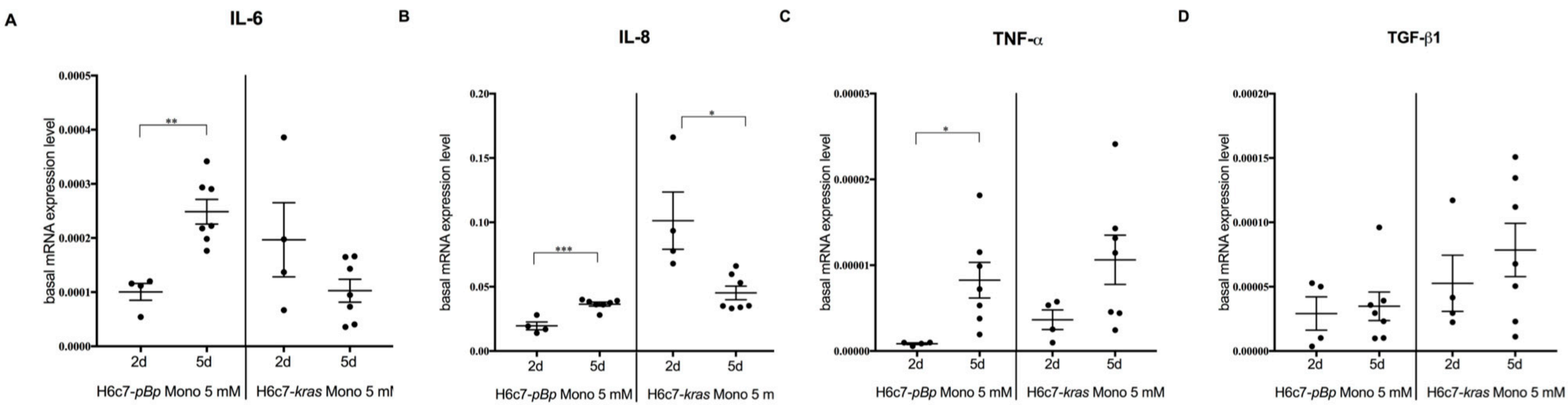

Figure S1: Basal mRNA levels of EMT- and CSC-inducers in benign and premalignant PDEC. H6c7-*pBp* or H6c7-*kras* cells were monocultured for 2 or 5 days under normoglycaemic conditions (5 mM D-glucose). RNA was isolated and analysed by qRT-PCR analysis. The relative mRNA levels of IL-6 (A), IL-8 (B), TNF-α (C) and TGF-β1 (D) are depicted, normalized to the housekeeping gene GAPDH and presented as basal mRNA expression level. Data are presented as mean and standard error of mean.

*: 0,05>p>0,0332 **: =0,0331 > p> 0,0021; ***=: 0,002 > p> 0,0001; n= 4 for 2-day culture, n=7 for 5-day culture
