# Supplementary material for: Initiation of Pancreatic Cancer: The Interplay of Hyperglycemia and Macrophages Promotes the Acquisition of Malignancy-Associated Properties in Pancreatic Ductal Epithelial Cells
Source: Int J Mol Sci. 2021 May 11;22(10):5086. doi: 10.3390/ijms22105086 (PMC8151031; doi:10.3390/ijms22105086)
Supplement: Supplementary file 1 [file ijms-22-05086-s001.zip › Figure S4.pdf]

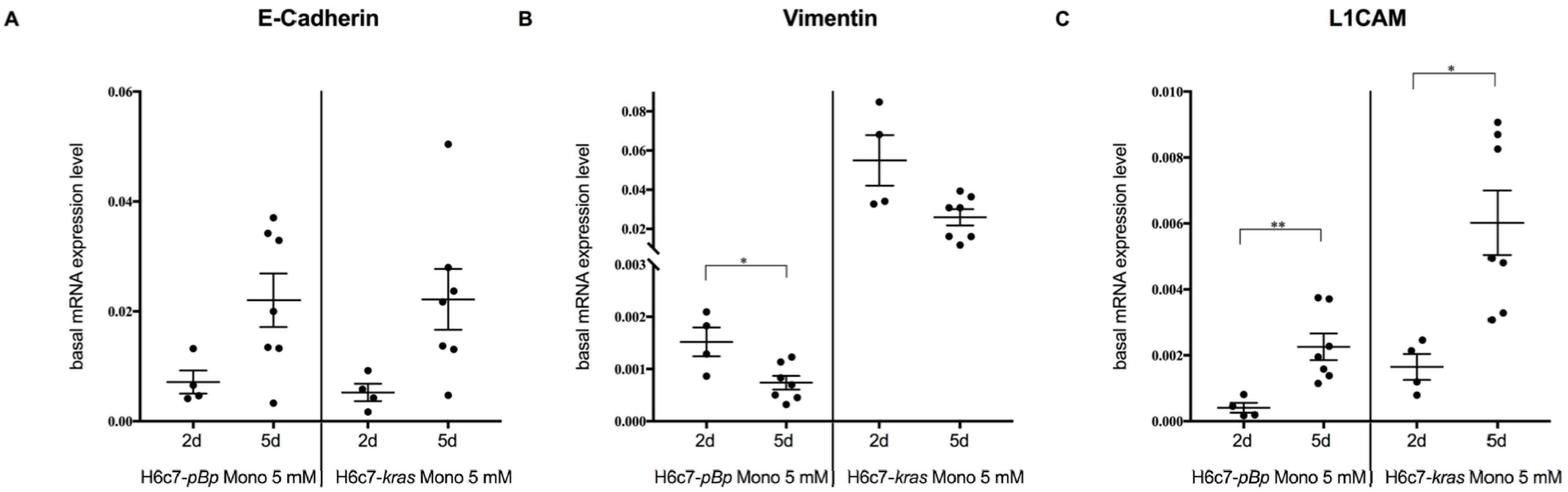

Figure S1: **Basal mRNA levels of EMT-marker in benign and premalignant PDEC.** H6c7-*pBp* or H6c7-*kras* cells were monocultured for 2 or 5 days under normoglycaemic conditions (5 mM D-glucose). RNA was isolated and analysed by qRT-PCR analysis. The relative mRNA levels of E-cadherin (A), L1CAM (B) and Vimentin(C) are depicted, normalized to the housekeeping gene GAPDH and presented as basal mRNA expression level. Data are presented as mean and standard error of mean. *: 0,05>p>0,0332 **: =0,0331 > p> 0,0021; ***=: 0,002 > p> 0,0001; n= 4 for 2-day culture, n=7 for 5-day culture
