# Supplementary material for: Initiation of Pancreatic Cancer: The Interplay of Hyperglycemia and Macrophages Promotes the Acquisition of Malignancy-Associated Properties in Pancreatic Ductal Epithelial Cells
Source: Int J Mol Sci. 2021 May 11;22(10):5086. doi: 10.3390/ijms22105086 (PMC8151031; doi:10.3390/ijms22105086)
Supplement: Supplementary file 1 [file ijms-22-05086-s001.zip › Figure S5.pdf]

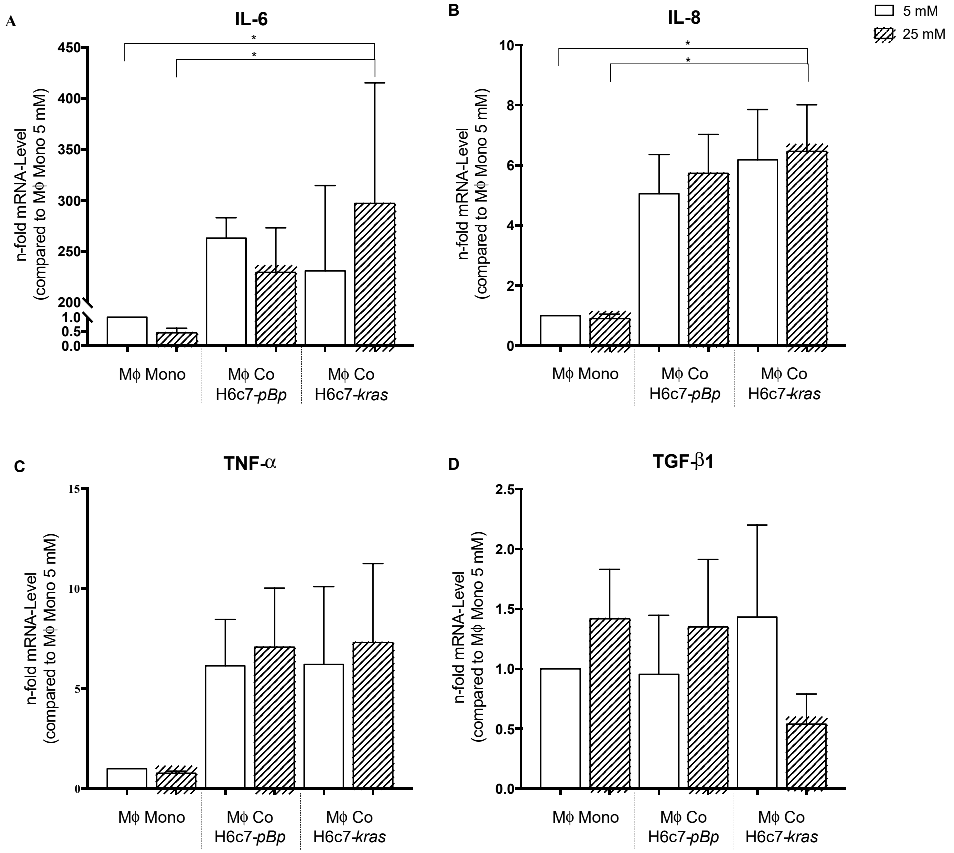


**Figure S5**: **The presence of benign and premalignant PDEC and high glucose levels promotes the pro- inflammatory phenotype of M1-polarized macrophages (MΦ).** M1-polarized macrophages were cultivated in mono- or coculture with H6c7-*pBp* or H6c7-*kras* cells for 5 days under normo- or hyperglycaemic conditions (5 or 25 mM D-glucose). MΦ were separated from direct coculture with epithelial cells via EpCam-MACS depletion of PDEC or harvested from monoculture and used for qRT-PCR analysis. The relative mRNA levels of IL-6 (A), IL-8 (B), TNF-α (C) and TGF-β1 (D) are depicted, normalized to the housekeeping gene GAPDH and presented as n-fold expression compared to the normoglycaemic monocultured MΦ sample. Data are presented as mean and standard error of mean. * :0,05>p>0,0332; n=5
