# Supplementary material for: Initiation of Pancreatic Cancer: The Interplay of Hyperglycemia and Macrophages Promotes the Acquisition of Malignancy-Associated Properties in Pancreatic Ductal Epithelial Cells
Source: Int J Mol Sci. 2021 May 11;22(10):5086. doi: 10.3390/ijms22105086 (PMC8151031; doi:10.3390/ijms22105086)
Supplement: Supplementary file 1 [file ijms-22-05086-s001.zip › Figure S6.pdf]

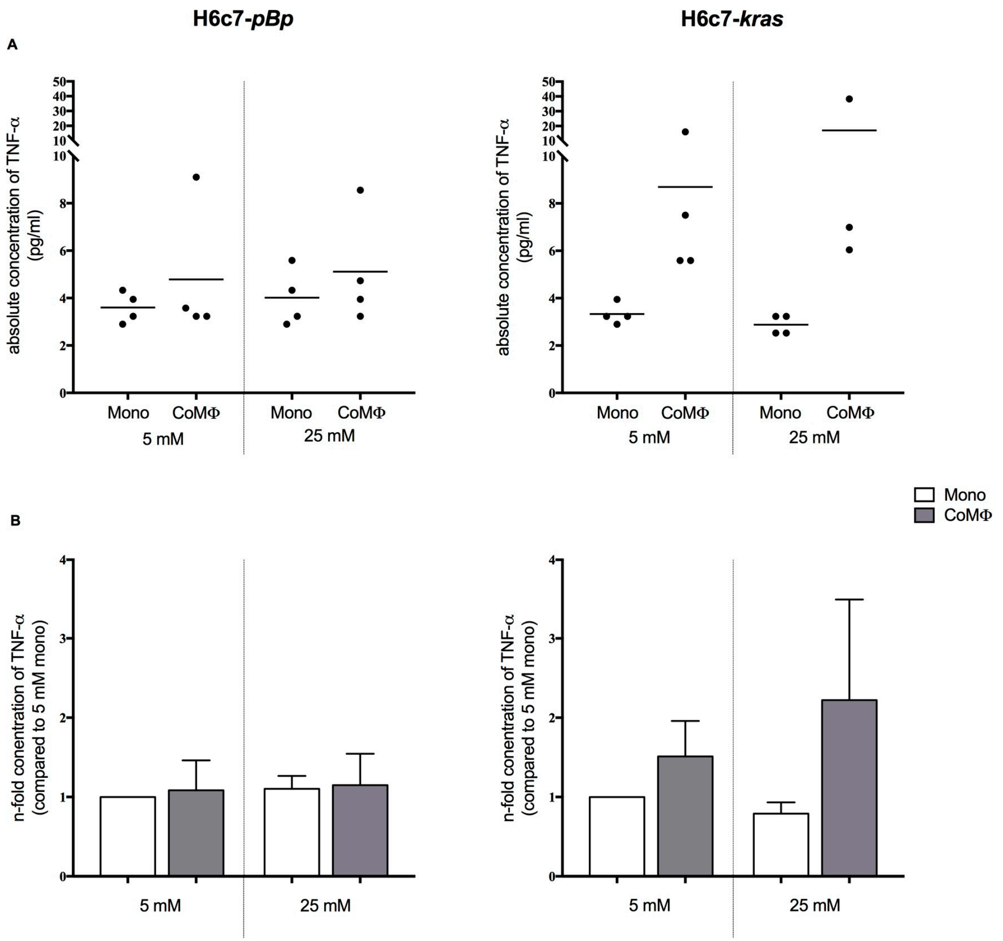

Figure S6. **TNF-α levels are elevated in the supernatants of premalignant PDEC upon exposure to macrophages and hyperglycaemia.** H6c7-*pBp* or H6c7-*kras* cells were cultivated in mono- or coculture with M1-polarized macrophages (MΦ) under normo- or hyperglycaemic conditions (5 or 25 mM of D-glucose) for 5 days. The supernatant was aspirated and used for Multiplex analysis. The absolute (A) and relative (B) concentration of TNF-α in the supernatants of the differing culture conditions is depicted. It is normalized to the cell number and presented as n-fold expression compared to the monocultured 5 mM sample. Normally distributed data are presented as mean and standard error of mean; n= 4
